# HuH-7 reference genome profile: complex karyotype composed of massive loss of heterozygosity

**DOI:** 10.1007/s13577-018-0212-3

**Published:** 2018-05-17

**Authors:** Fumio Kasai, Noriko Hirayama, Midori Ozawa, Motonobu Satoh, Arihiro Kohara

**Affiliations:** Japanese Collection of Research Bioresources (JCRB) Cell Bank, National Institutes of Biomedical Innovation, Health and Nutrition, Saito-Asagi 7-6-8, Ibaraki, Osaka 567-0085 Japan

**Keywords:** Genome instability, Tumor cell line, Heterogeneity

## Abstract

Human cell lines represent a valuable resource as in vitro experimental models. A hepatoma cell line, HuH-7 (JCRB0403), has been used extensively in various research fields and a number of studies using this line have been published continuously since it was established in 1982. However, an accurate genome profile, which can be served as a reliable reference, has not been available. In this study, we performed M-FISH, SNP microarray and amplicon sequencing to characterize the cell line. Single cell analysis of metaphases revealed a high level of heterogeneity with a mode of 60 chromosomes. Cytogenetic results demonstrated chromosome abnormalities involving every chromosome in addition to a massive loss of heterozygosity, which accounts for 55.3% of the genome, consistent with the homozygous variants seen in the sequence analysis. We provide empirical data that the HuH-7 cell line is composed of highly heterogeneous cell populations, suggesting that besides cell line authentication, the quality of cell lines needs to be taken into consideration in the future use of tumor cell lines.

**Electronic supplementary material:**

The online version of this article (10.1007/s13577-018-0212-3) contains supplementary material, which is available to authorized users.

## Introduction

Human cancer cell lines have been widely used in in vitro experiments to study tumor biology and to develop new drugs [1]. Cancer cells usually have a different genome structure from normal [2], therefore, genomes of human cell lines cannot be fully explained by the human reference genome [3]. Because each cell line has unique features, characterization of tumor cell lines is required to evaluate the similarities and differences that exist in genomic features between cell lines and clinical samples. Different from the limited availability of tumor tissue samples, cancer cell lines play an important role in maintaining a persistent cellular resource.

The HuH-7 cell line was established in 1982 from a well differentiated human hepatocellular carcinoma [4]. This cell line has been characterized by the production of a variety of physiologically active substances including alpha-fetoprotein and albumin [5, 6]. It is highly susceptible to the hepatitis C virus (HCV) and is used for an HCV replicon system [7], allowing production of infectious HCV particles in vitro and permitting the development of drugs against HCV. HuH-7 is a well-known cell line used as a model for investigating both hepatoma and HCV.

Tumor cells acquire genetic alterations during their evolution, which underlie cancer development, progression and drug resistance [8]. It should be noted that genome profiles of tumor cell lines are not always identical between different passages under the same name [9]. Because clonal evolution of tumor cells in vitro is different from in vivo [9], heterogeneity in cell lines can be changed during cell culture [10]. It is reported that HCV replication using HuH-7 cells was possible only in certain sub-population [11]. This indicates that this cell line consists of heterogeneous cell populations, which could cause differences in genome profiles between laboratories.

Although the HuH-7 cell line has been in regular use for over 25 years, an accurate genome profile has not yet been established. In this study, genetic analyses based on karyotyping, SNP microarray and variant calls on major cancer-related genes were performed to provide a reference standard.

## Materials and methods

### Cell culture conditions and DNA extraction

The HuH-7 cell line has been registered at the JCRB cell bank as JCRB0403 and is distributed worldwide upon request. The culture medium was DMEM with l-glutamine, low glucose (1 g/L) and 10% non-heat-inactivated fetal bovine serum without antibiotics. Cells were treated with 0.25% trypsin and 0.02% EDTA, and split at 1/4 dilution. Our standard quality control confirmed that samples were free of mycoplasma and major pathogenic human viruses. Genomic DNA was extracted from cultured cells at passage 49 using the AllPrep DNA/RNA Mini Kit (Qiagen).

### Cell line authentication

The DNA sample was amplified by the PowerPlex 16 STR System (Promega) and repeat numbers were determined by the ABI 3500 Genetic Analyzer.

### Metaphase chromosome analysis

Metaphase chromosomes were prepared from cells at passage 57 using a conventional protocol [12]. Chromosome numbers were counted on metaphases stained with Giemsa. Multi-color fluorescence in situ hybridization (M-FISH) was performed according to the manufacturer’s protocol (24 × Cyte kit MetaSystems). Signal detection and subsequent analysis of metaphases were carried out using the Metafer system and Isis software (Metasytems).

### Whole genome analysis based on SNP microarray

DNA microarray analysis was performed using a high-density chip, CytoScan HD array (Affymetrix). The data analysis was undertaken using the Chromosome Analysis Suite software (Affymetrix).

### Mutation analysis

Target regions were amplified using the Ion AmpliSeq Cancer Hotspot Panel v2 (Life Technologies). Template DNA was prepared using the Ion PGM Hi-Q Chef Kit (Life Technologies) and sequencing was run on the Ion PGM using the Ion 314 chip. Reads were aligned to the hg19 reference and the analysis was carried out using the Ion Torrent Variant Caller Plugin and the Ion Reporter (Life Technologies).

### Flow cytometry

Expression of cell surface markers was examined at passage 50 by flow cytometry with a standard protocol [12] using eight antibodies and their isotype controls listed in Table S1.

## Results

### Cell culture and cell morphology

HuH-7 cells showed epithelial morphology and some of them contain small droplets in the cytoplasm (Figure S1). The optimal seeding cell density at passage 45–50 was 1–2 × 10^4^ cells/cm^2^, and the saturation density was about 6 × 10^4^ cells/cm^2^. The doubling time was calculated to be 1.5–2.0 days. Low seeding density or insufficient inhibition of trypsin activity during subculture tended to cause growth retardation.

### STR profile

An STR profile of 16 loci is shown in Table S2, which indicates the absence of Y chromosome. This consists of one pattern for 11 loci and two patterns for five loci, which is unique in that the number of homozygous loci is more than that of heterozygous loci.

### Chromosome number

**Fig. 1:**
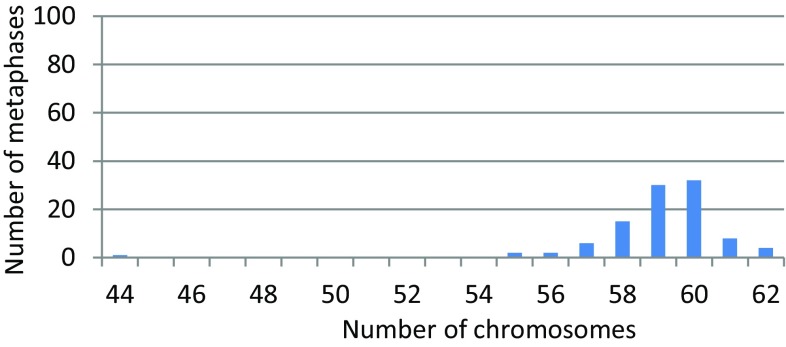
Chromosome number examined in 100 Giemsa-stained metaphases, showing the modal number of 60 chromosomes. The peak is not significant and chromosome numbers are varied between cells, indicating heterogeneous cell populations

The majority of cells showed a chromosome number between 55 and 63 (Fig. 1). The mode at 60 chromosomes consisted of less than 1/3 of the population, indicating heterogeneous cell populations.

### Karyotyping by M-FISH

**Fig. 2:**
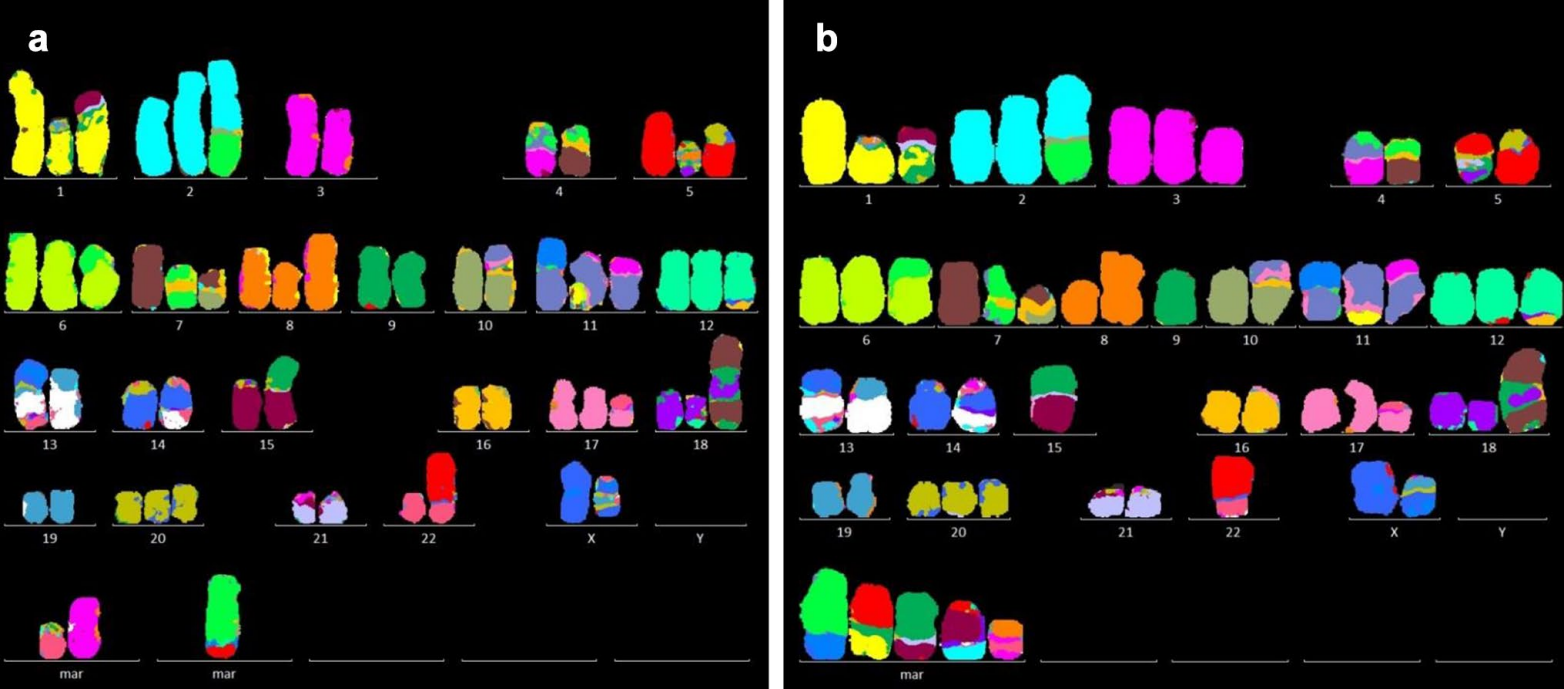
Examples of M-FISH karyograms from two major clones in the HuH-7 cell line. Chromosomes labeled by a single color correspond to either a normal chromosome or have intra-chromosomal rearrangements. Chromosomes painted by two or more colors indicate inter-chromosome rearrangements. M-FISH cannot detect cryptic inter- and intra-chromosomal changes which resulted in the original size. Common abnormalities are aligned with a standard order as normal chromosomes. Subline- or clone-specific aberrations are captured and displayed at the bottom. **a** A karyotype with 60 chromosomes has two subline- and one clone-specific abnormalities. Every chromosome involves rearrangements, however, one or two chromosomes in each pair remain apparently normal, except for chromosomes 2, 4, 11, 13 and X in which both demonstrate rearrangements. **b** Another karyotype with 58 chromosomes has five additional changes specific for this subline. The full description of chromosome abnormalities and the original images in each karyotype are shown in Figure S2

Abnormalities were detected in all chromosomes except for chromosome 21, which was an apparently normal chromosome pair (Fig. 2). M-FISH revealed 32 common abnormalities consisting of trisomy 20, loss of Y, 6 partial intra-chromosomal gains or losses, and 24 inter-chromosomal rearrangements. However, analysis of 22 metaphases showed 20 different patterns, indicating highly heterogeneous cell populations in this cell line. Although the most basic clone was not detected, karyotypes were classified into one group of subclones close to the stemline and two groups of sidelines. Because no karyotype corresponding to a main clone was observed, the HuH-7 cell line was described as composite karyotype without a stemline. The HuH-7 karyotype could be described using ‘idem’ for common abnormalities by the simple expedient as follows.

58 ~ 62,der(X)t(X;14),+der(X)t(X;19),-Y,del(1),+der(1)t(1;15),del(2),der(2)t(2;2),+der(2)t(2;4),del(3),+del(3),der(4)t(3;4),+der(4)t(4;7),der(5)t(5;8;18),+der(5)t(5;20),+der(6)t(4;6),der(7)t(4;7),+der(7)t(7;10),del(8),+dup(8),der(10)t(8;10;11),der(11)t(X;11),der(11)t(1;11),+der(11)t(3;11),+der(12)t(12;16),der(13)t(X;13),der(13)t(13;19),der(14)t(13;14),der(15)t(9;15),+der(17)t(17;22),del(18),+der(18)t(7;18),+20,der(22)t(5;22),+der(22)t(7;22)[cp10]/59,idem,-del(3),+der(4)t(X;4)[cp4]/57 ~ 59,idem,-del(3),-der(22),+der(4)t(X;4),der(5)t(1;5),der(9)t(9;15),der(15)t(2;15),der(22)t(8;22)[cp8].

### SNP microarray

**Fig. 3:**
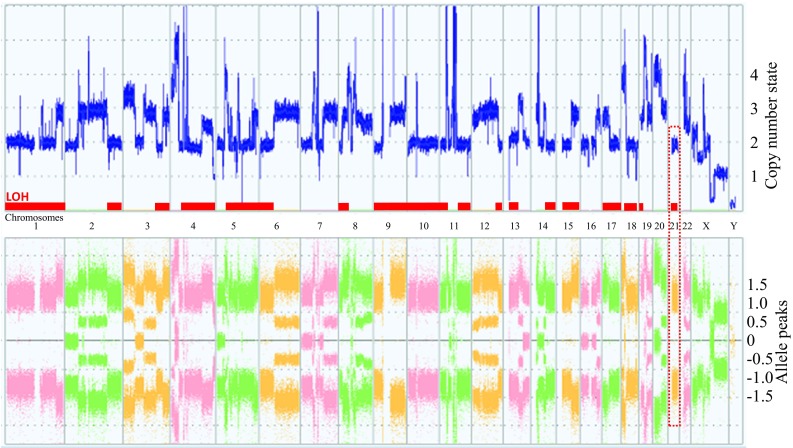
A whole genome profile based on an SNP-based microarray show copy number (upper) and allele (lower) profiles. Partial copy number gains are detected across the whole genome. Copy number state shows two normal copies, however, the allele patterns consist of two peaks indicating the copy neutral LOH shown by the red dashed square. Allele peaks present  between − 0.5 and 0.5 indicate heterozygous loci, implying that other regions are composed of LOH indicated by red lines

Major copy number changes showed regional gains except for a complete deletion at 5q21.3and at Xq21.1 (Fig. 3). In contrast to the chromosomes 7, 16, 20 and 22; composed of two different alleles, the entire chromosomes 1, 9, 10, 15, 17 and 21 presented one SNP allele type, showing a loss of heterozygosity (LOH) (Figure S3). The total number of LOH regions extended to 1680.378 Mb, accounting for 55.3% of the human genome based on the hg19 reference. Although M-FISH and the copy number profile showed an apparently normal chromosome 21, the SNP allele profile revealed a copy neutral LOH. A highly complex regional profile was detected at 11q12–14, indicating the occurrence of chromothripsis. The mosaic copy number observed at 4q, 22q and Xp implies the difference in genome between cells, corresponding to chromosome changes in the sideline (Fig. 2b). The absence of the Y chromosome would be due to the entire loss that is frequently observed in male tumor cells.

### Mutation profile

Amplicon sequences for 207 amplicon Variants were detected at seven positions with frequencies of 100%, involving six genes (Table 1). Of these, four variants are synonymous substitutions and others are missense mutations. One, TP53 p.Tyr220Cys, is reported in the COSMIC database.

**Table 1:** Variant profiles based on AmpliSeq™ Cancer Hotspot Panel

Locus	Ref.	Observed allele	Genes	Variant ID	% Frequency	Exon	Coding	Amino acid change	Variant effect
chr4:1807894	G	A	FGFR3		100	14	c.1959G>A	p.(=)	Synonymous
chr4:55141050	AGCCCA	AGCCCG	PDGFRA	COSM12417	100	12	c.1701A>G	p.(=)	Synonymous
chr4:55972974	T	A	KDR		100	11	c.1416A>T	p.Gln472His	Missense
chr5:112175769	CGG	CAG	APC	COSM19714	100	16	c.4479G>A	p.(=)	Synonymous
chr10:43613843	G	T	RET		100	13	c.2307G>T	p.(=)	Synonymous
chr17:7578190	T	C	TP53	COSM10758	100	6	c.659A>G	p.Tyr220Cys	Missense
chr17:7579472	G	C	TP53	COSM45985	100	4	c.215C>G	p.Pro72Arg	Missense

### Cell surface markers

Flow cytometry analysis of HuH-7 cells detected the expression of CD24, CD133 and EpCAM (Figure S4), which were consistent with previous studies [13, 14]. CD44 was moderate, in contrast to the absence of CD90 and CD95. Very low levels of CD90 expression were reported [15], indicating the presence of positive cells in subclonal populations. Loss of CD95 expression is associated with mutations in p53 [16], which can be observed in this study.

## Discussion

The variable chromosome number in the HuH-7 cell line indicates cellular heterogeneity. This is observed in its diverse karyotypes, implying the presence of clonal variants. The cell population could become heterogeneous due to selective pressure during culture [9]. This would be influenced by culture conditions, which can differ between laboratories [17]. Although a previous study showed an example of a G-banding karyotype of the HuH-7 cell line, without specifying the source of the sample, the complex rearrangements were not accurately determined [18]. Although the reference karyotype cannot be represented by one metaphase, common chromosomal abnormalities identified by M-FISH can be used as cytogenetic markers for the HuH-7 cells despite the extreme heterogeneity.

Karyotypes including chromosome rearrangements clearly exhibit differences between normal and abnormal homologues [19]. Analysis using total genomic DNA does not distinguish differences between homologues, however, this can be achieved by chromosome sequencing [20]. As long as DNA samples are prepared from the bulk of cells, it is difficult to characterize heterogeneous samples by microarray or sequencing techniques [21]. In addition, it is hardly possible to construct karyotypes with multiple rearrangements by these approaches. Chromosome analysis of individual metaphases corresponds to a single cell basis which reveals differences between heterogeneous cells. Karyotyping data are a robust method of investigating heterogeneous cell populations and lead to a better understanding of tumor genomes when combined with other results which demonstrate sequence variants or copy number changes.

Whole genome sequencing provides a picture of nucleotide composition at the highest resolution; however, the data quality largely depends on the techniques and equipment available [22]. Sequence data from a large number of human cancer cell lines have been accumulated in the Cancer Cell Line Encyclopedia [23] and the Catalogue of Somatic Mutations in Cancer [24], but discrepancies between these two databases have been reported [25]. This could be due to the admixture of heterogeneous cells occurring during cell line evolution, indicating that heterogeneity causes difficulties in the analysis of tumor samples.

Loss or gain of a whole chromosome is due to chromosome mis-segregation arising through defects in the mitotic checkpoint [26]. Mutations related to these functions might have occurred in the early stages of tumor formation, so that HuH-7 cells can acquire the potential for continuous genome evolution. Because LOH is observed across the HuH-7 genome, including rearranged chromosomes, whole chromosome changes might have occurred prior to rearrangements. Copy neutral LOH is caused mostly by the loss of the wild type allele, leading to the selection of deleterious mutations [27]. This indicates that a high level of LOH could result in a high frequency of chromosome rearrangements, implying that the HuH-7 genome can be easily changed during cell culture.

Expression of CD133, known as a cancer stem cell marker, has been reported in a sub-population of the HuH-7 cell line [28]. It is suggested that cancer cell lines contain a sub-population of cancer stem-like cells [29, 30]. They have the potential to undergo self-renewing divisions, express stem cell markers and exhibit increased tumorigenicity [29], which can remain in culture of tumor cell lines [31]. Because cell lines are maintained through subculture, which involves dilutions of cells and causes the expansion of certain clones with higher growth advantages, it is unlikely that founder clones in tumor cell lines can remain through serial passages [9]. This implies that cancer stem cells in tumor cell lines would undergo in vitro clonal evolution and could have different genome profiles between passages.

Quality control of cell lines is essential to obtain consistent and reproducible data, which is important when making comparisons with previous studies or databases [32]. Although analysis of the same cell lines with the same methodology is expected to show the same results between different laboratories, discrepancies between studies are found in drug response phenotypes, which could be caused by phenotypic differences in cell lines [33]. Human cell line authentication is established based on STR analysis, but this method can only identify the origin of cells and cannot distinguish cell lines of the same origin [34]. Because most cancer cells are genetically unstable and undergo progressive rearrangements, quality of cell lines largely depends on culture history including passage numbers and results from management of cell culture [35]. Our study shows the fundamental features of the HuH-7 cell line, providing empirical evidence for risk factors in the use of tumor cell lines.

## Electronic supplementary material

Below is the link to the electronic supplementary material.

Supplementary material 1 (PDF 896 KB)
