# Supplementary material for: HuH-7 reference genome profile: complex karyotype composed of massive loss of heterozygosity
Source: Hum Cell. 2018 May 17;31(3):261–7. doi: 10.1007/s13577-018-0212-3 (PMC6002425; doi:10.1007/s13577-018-0212-3)
Supplement: Supplementary file 1 — Supplementary material 1 (PDF 896 KB) [file 13577_2018_212_MOESM1_ESM.pdf]

Figure S1

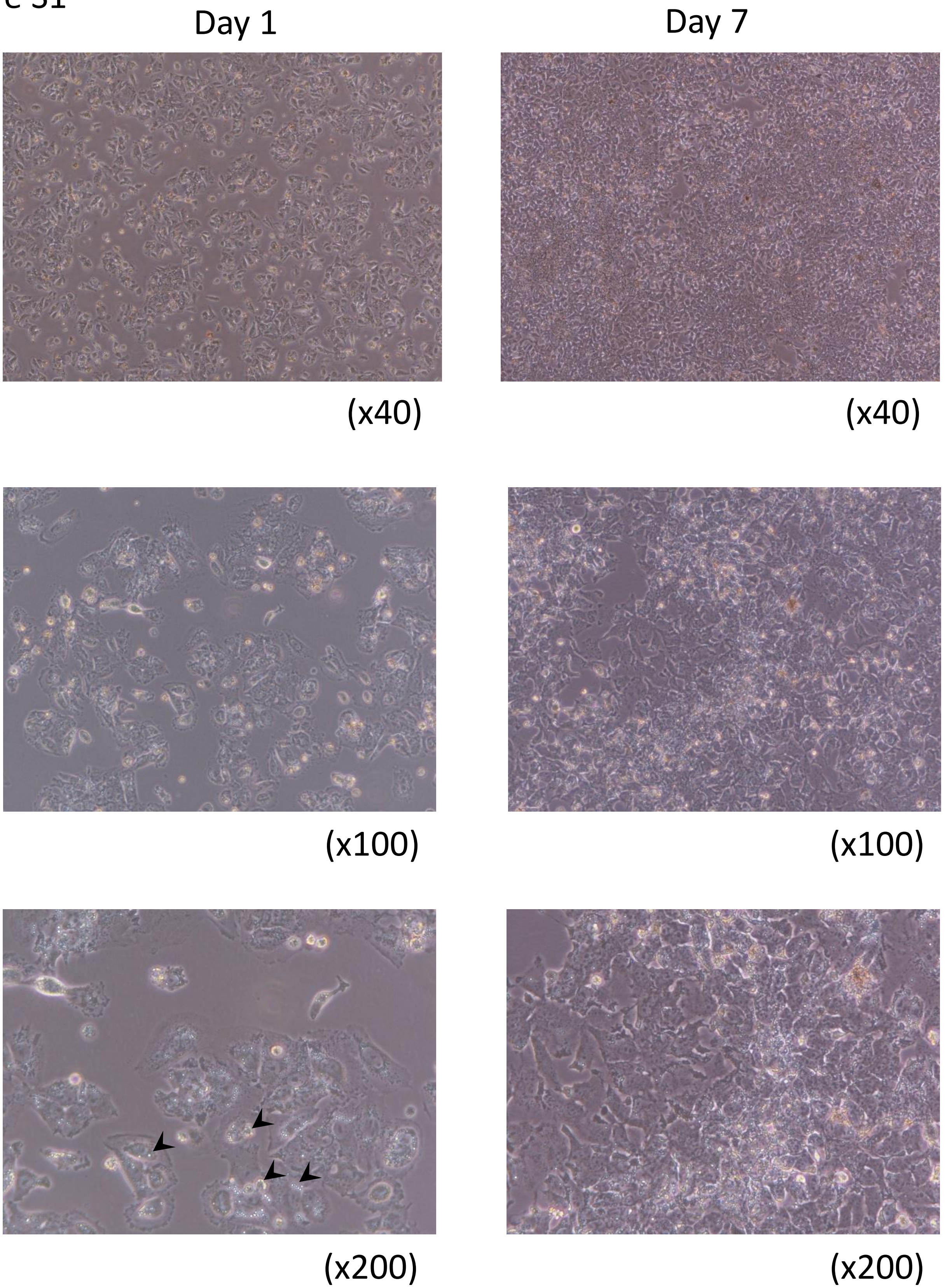

The morphology of HuH-7 cells in culture. Left; the culture 1 day after seeding. Right; after 7 days of culture, the cells became about 90% confluent. HuH-7 cells formed epithelial-like monolayer. Some cells contain droplet-like structure (arrowheads).

Figure S2

A

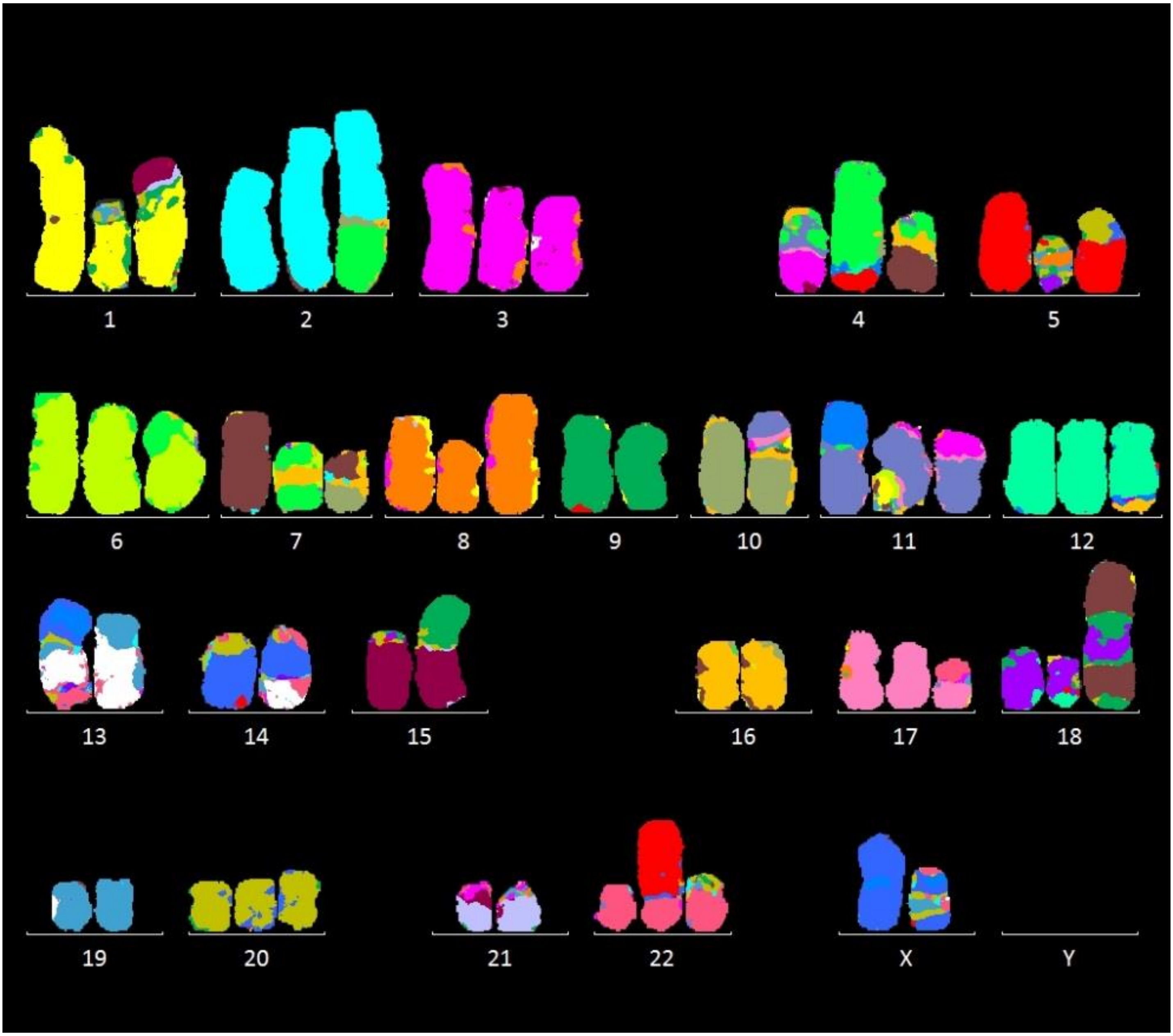

Karyotype of Figure 2A
60,der(X)t(X;14),+der(X)t(X;19),-Y,del(1),+der(1)t(1;15),del(2),der(2)t(2;2),+der(2)t(2;4),del(3),+del(3), der(4)t(3;4),*der(4)t(4;5)*,+der(4)t(4;7),der(5)t(5;8;18),+der(5)t(5;20),+der(6)t(4;6), der(7)t(4;7),+der(7)t(7;10),del(8),+dup(8),der(10)t(8;10;11),der(11)t(X;11), der(11)t(1;11),+der(11)t(3;11), +der(12)t(12;16),der(13)t(X;13),der(13)t(13;19),der(14)t(13;14),der(15)t(9;15),+der(17)t(17;22),del(18),+ der(18)t(7;18),+20,der(22)t(5;22),+der(22)t(7;22)

Figure S2

B

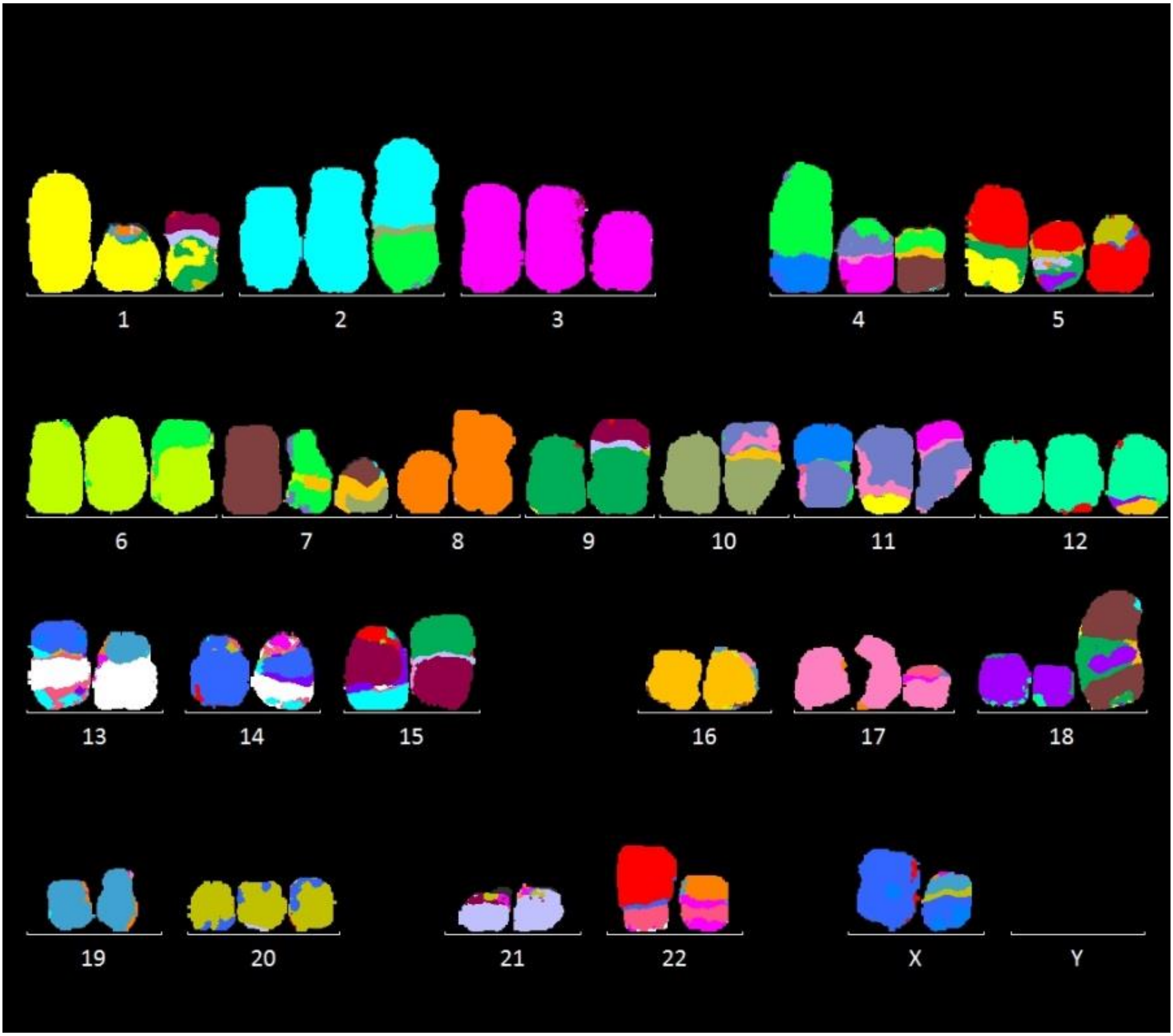

Karyotype of Figure 2B

58,der(X)t(X;14),+der(X)t(X;19),-Y,del(1),+der(1)t(1;15),del(2),der(2)t(2;2),+der(2)t(2;4),+del(3), der(4)t(3;4),der(4)t(4;7),+der(4)t(X;4),der(5)t(1;5),der(5)t(5;8;18),der(5)t(5;20),+der(6)t(4;6), der(7)t(4;7),+der(7)t(7;10),del(8),+dup(8),der(9)t(9;15),der(10)t(8;10;11), der(11)t(X;11),der(11)t(1;11),+der(11)t(3;11),+der(12)t(12;16),der(13)t(X;13),der(13)t(13;19), der(14)t(13;14),der(15)t(2;15),der(15)t(9;15),+der(17)t(17;22),del(18),+der(18)t(7;18),+20, der(22)t(5;22),der(22)t(8;22)

Figure S2

C

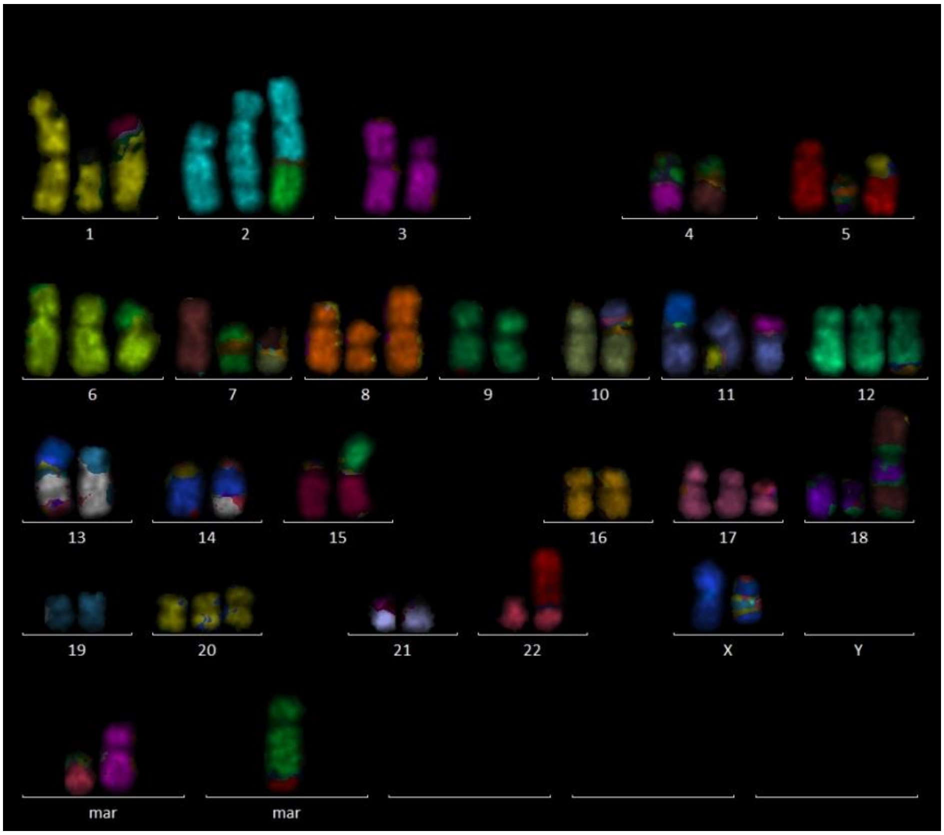

D

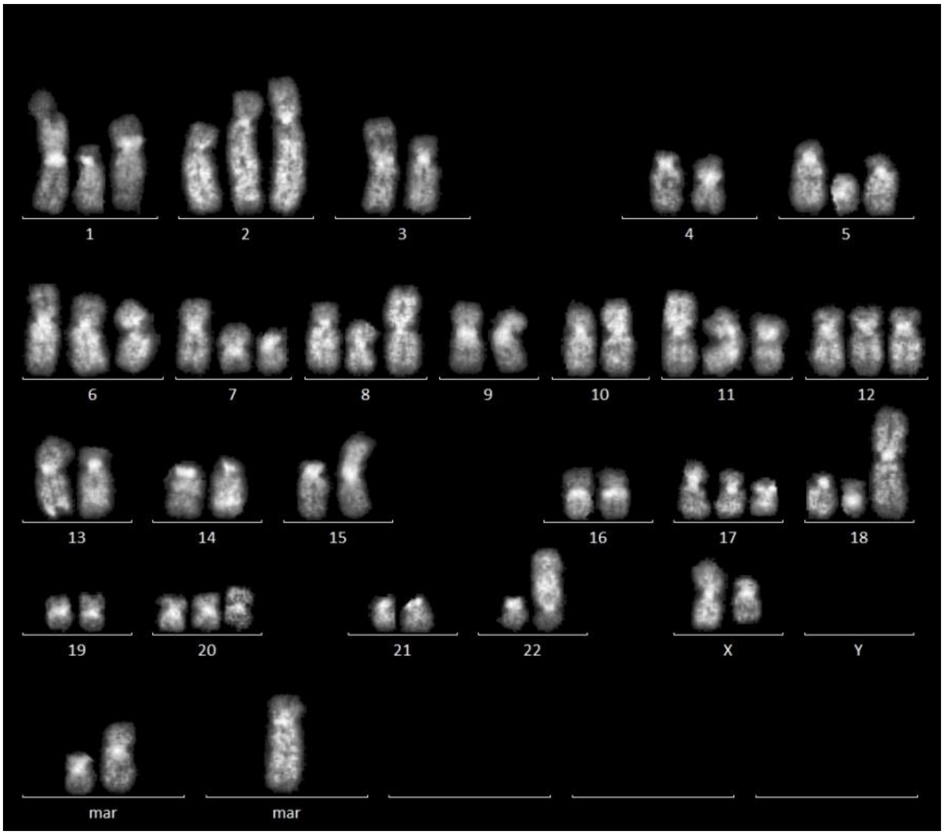

The original M-FISH (C) and DAPI (D) images of Figure 2A.

Figure S2

E

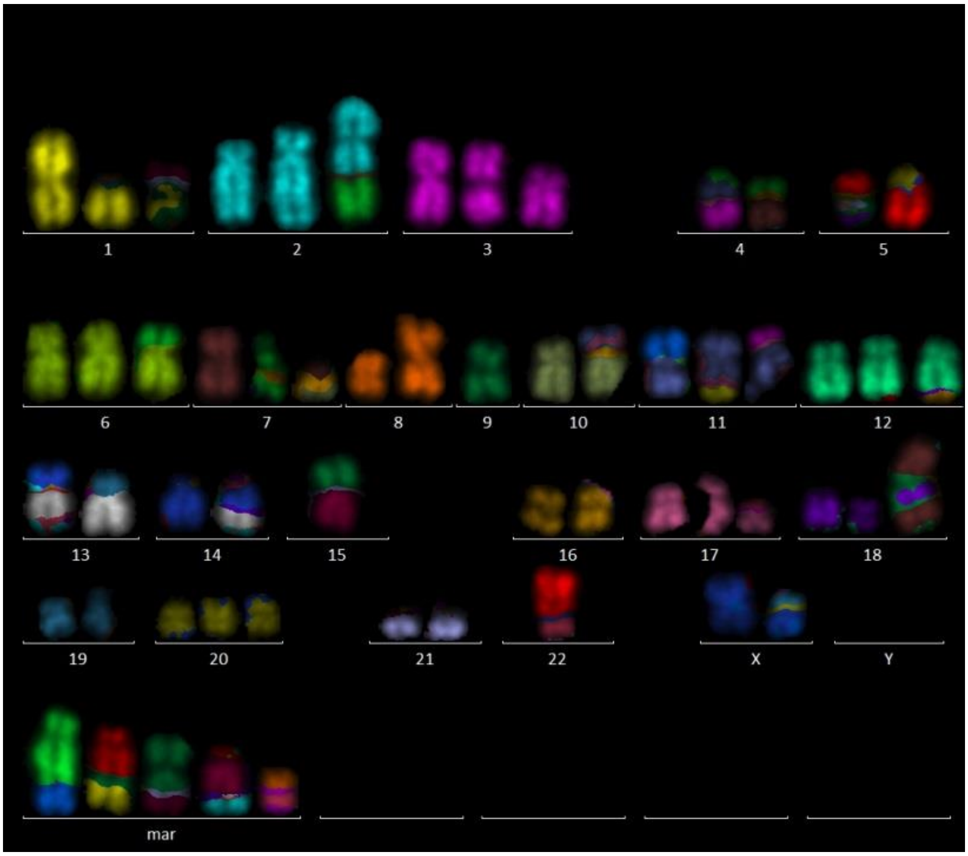

F

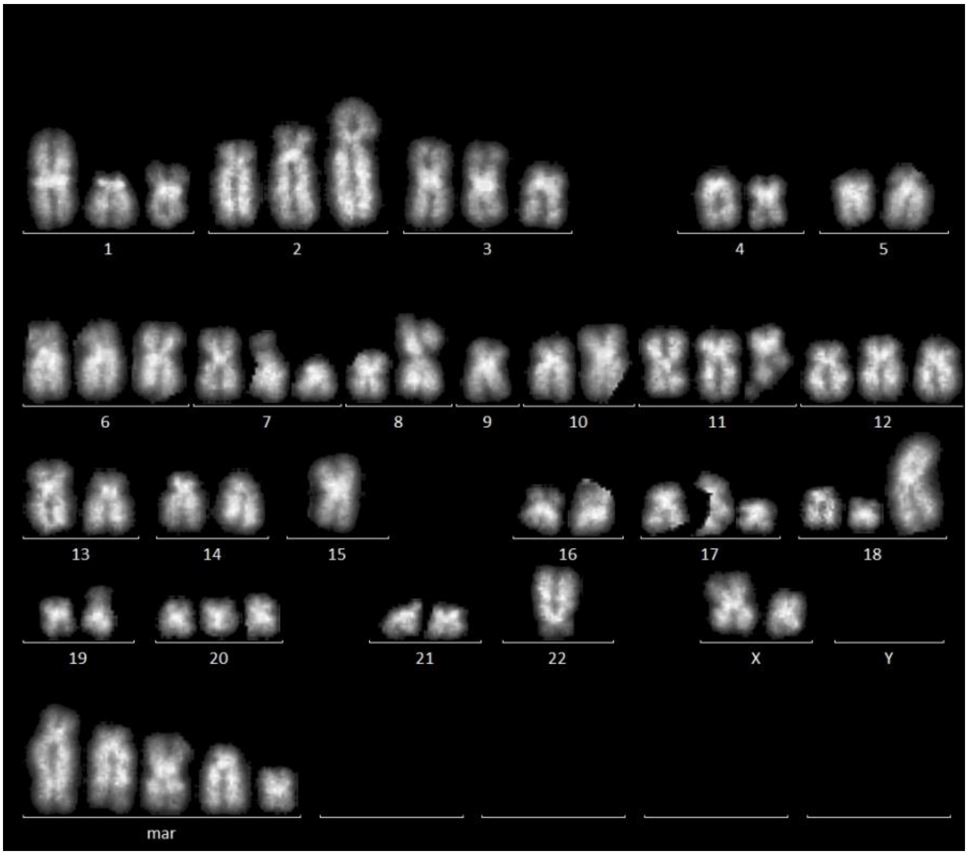

The original M-FISH (E) and DAPI (F) images of Figure 2B.

Figure S3

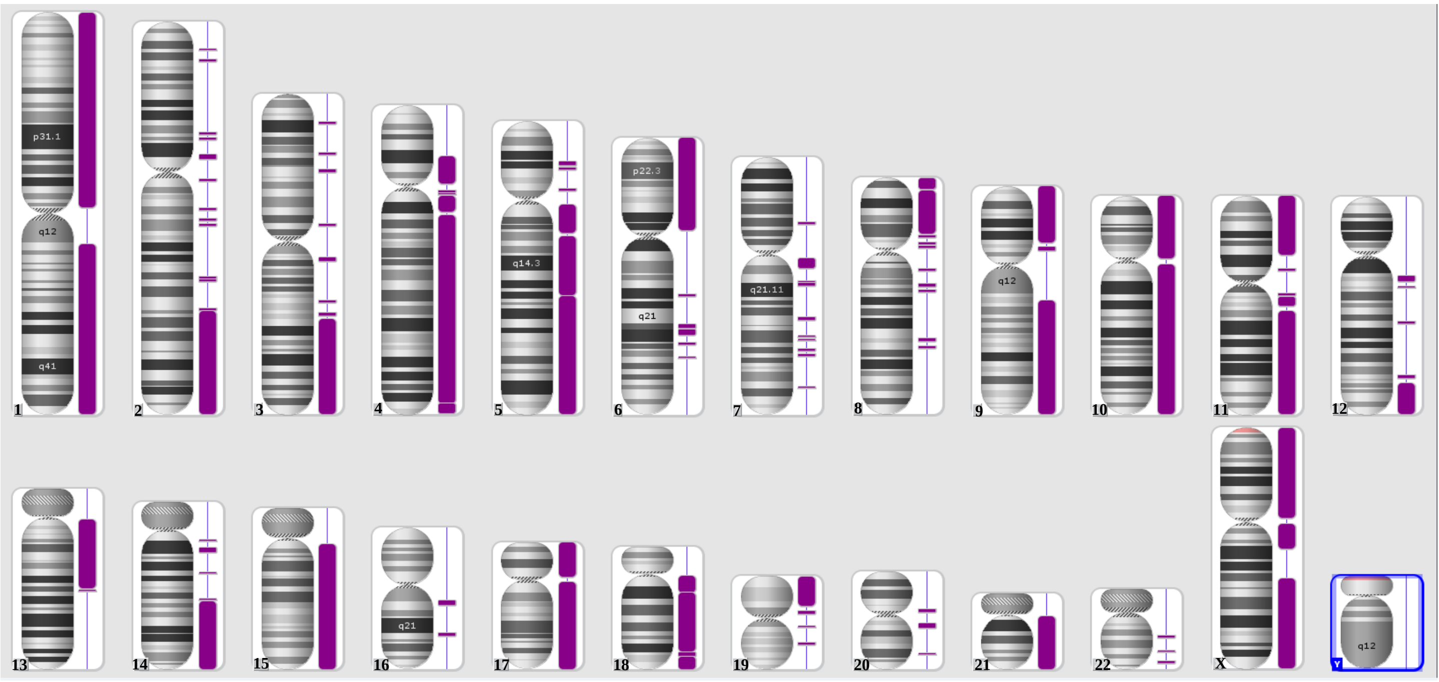

LOH regions identified by SNP microarray. LOH is observed in entire chromosomes 1, 9, 10, 15, 17 and 21. Others exhibit LOH in a part of chromosomes except for chromosomes 7, 16, 20 and 22. LOH of X chromosome corresponds to heterozygous sex chromosomes in the normal cells because of the male origin.

Figure S4

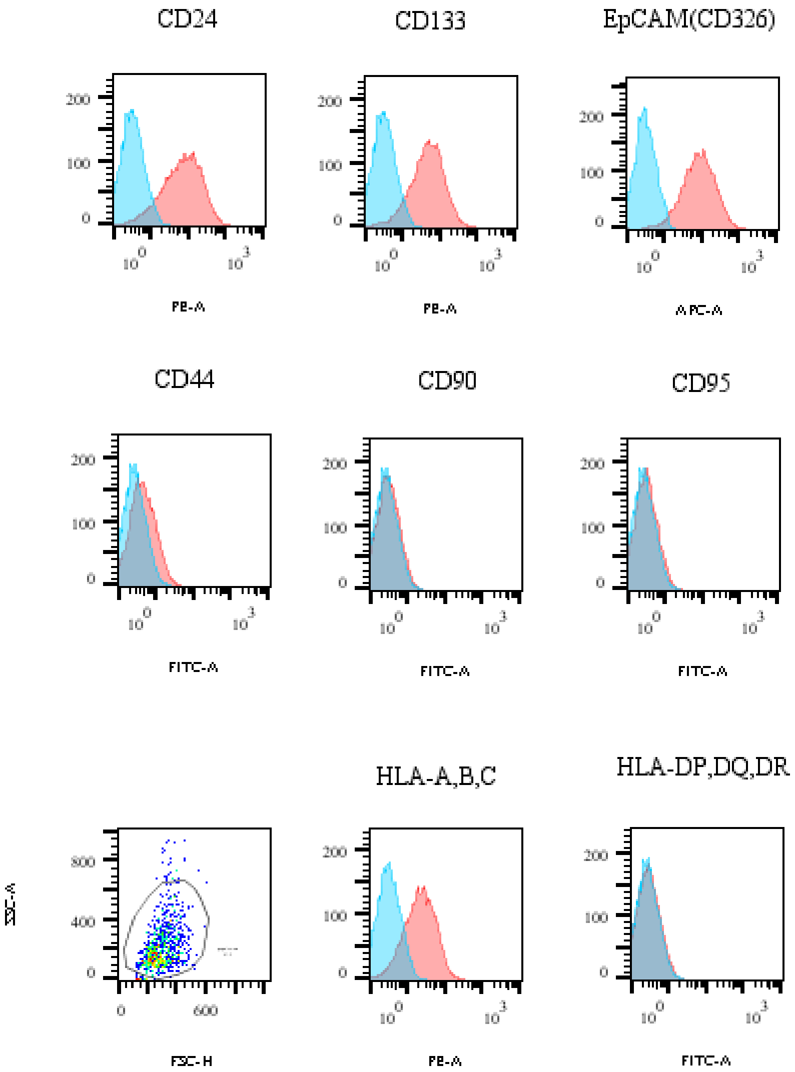

Flow cytometry analysis of expression of cell surface antigen molecules on HuH-7. Expression of CD24, CD133 and EpCAM were clearly detected compared to lower level of CD44. CD90 and CD95 were undetectable. HLA-A,B,C and HLA-DP,DQ,DR were used for positive and negative controls, respectively.

Table S1

Cell surface markers used for flow cytometry

cell surface antigen	Isotype	fluorescent labeling	manufacturer, cat. No.	Isotype control	fluorescent labeling	manufacturer, cat. No.
CD24	mouse IgG1	RPE	Miltenyi Biotec, 130-095-953	mouse IgG1/mouse IgG2a	FITC/RPE	DAKO, X0298
CD44	mouse IgG1	FITC	Beckman coulter, IM1219U	mouse IgG1	FITC	Beckman coulter, A10974
CD90	mouse IgG1	FITC	DAKO, F7274	mouse IgG1	FITC	Beckman coulter, A10974
CD95	mouse IgG1	FITC	Beckman coulter, IM1506	mouse IgG1	FITC	Beckman coulter, A10974
CD133	mouse IgG1	RPE	Miltenyi Biotec, 130-080-801	mouse IgG1	RPE	DAKO, X0298
EpCAM(CD326)	mouse IgG1	APC	Miltenyi Biotec, 130-091-254	mouse IgG1	APC	DAKO, X0968
HLA-A,B,C	mouse IgG2a	RPE	DAKO, R7000	mouse IgG1/mouse IgG2a	FITC/RPE	Beckman coulter, A10974
HLA-DP,DQ,DR	mouse IgG1	FITC	DAKO, F0817	mouse IgG1/mouse IgG2a	FITC/RPE	Beckman coulter, A10974

Table S2

STR profiles of 16 loci.

TPOX	D3S1358	FGA	D5S818	CSF1PO	D7S820	D8S1179	TH01	vWA	D13S317	Penta E	D16S539	D18S51	D21S11	Penta D	AMEL
2p25.3	3p21.31	4q31.3	5q23.2	5q32	7q21.11	8q24.13	11p15.5	12p13.31	13q31.1	15q26.2	16q24.1	18q21.3	21q21.1	21q22.3	Xp22.2
8, 11	15	22,23	12	11	11	14,15	7	16, 18	10, 11	11	10	15	30	12	X
